# Expression of the onconeural protein CDR1 in cerebellum and ovarian cancer

**DOI:** 10.18632/oncotarget.25252

**Published:** 2018-05-08

**Authors:** Cecilie Totland, Torbjørn Kråkenes, Kibret Mazengia, Mette Haugen, Christian Vedeler

**Affiliations:** ^1^ Department of Neurology, Haukeland University Hospital, Bergen, Norway; ^2^ Department of Clinical Medicine, University of Bergen, Bergen, Norway

**Keywords:** PCD, CDR1, Purkinje cells, ovarian cancer, autoantibodies

## Abstract

Cerebellar degeneration related protein 1 (CDR1) is expressed in the cerebellum, and CDR1 antibodies have been associated with paraneoplastic cerebellar degeneration (PCD). In this study, we examined CDR1 expression in cerebellum and in ovarian and breast tumors, as well as the intracellular localization of CDR1 in cancer cells in culture. CDR1 was strongly expressed in the cytosol and dendrites of Purkinje cells and in interneurons of the molecular layer in cerebellum. CDR1 was also present in ovarian and breast tumors, as well as in ovarian and breast cancer cell lines, but was not present in normal breast or ovarian tissue. In cells overexpressing CDR1, CDR1 localized close to the plasma membrane in a polarized pattern at one edge. CDR1 was strongly expressed on the outer surface, apparently in filopodias or lamellipodias, in cells endogenously expressing CDR1. Overexpression of CDR1 showed a 37 and a 45 kDa band in western blot. The 37-kDa isoform was present in 16 ovarian cancer lysates, while the 45-kDa isoform was only found in three ovarian cancer patients. The presence of CDR1 in ovarian cancer was not associated with PCD. CDR1 antibodies were only found in serum from one patient with PCD and ovarian tumor with metastases. Therefore, CDR1 is probably not a marker for PCD. However, CDR1 may be associated with cell migration and differentiation.

## INTRODUCTION

Paraneoplastic neurological syndromes (PNS) are rare disorders associated with different forms of cancer, most commonly small-cell lung cancer, ovarian cancer and breast cancer. Such tumors are more likely to overexpress onconeural proteins, and PNS occurs as an autoimmune response where autoantibodies and cytotoxic T-cells meant to target tumor antigens also attack neurons expressing similar proteins [1, 2]. Paraneoplastic cerebellar degeneration (PCD) is a severe form of PNS affecting a small subset of women with breast or ovarian cancer. PCD can be diagnosed by the presence of Yo antibodies, which are specific for cerebellar degeneration related proteins 2 and 2L (CDR2 and CDR2L) [3-5]. It has also been reported that Yo antibodies can react with CDR1 [6, 7].

In 1986 it was reported that sera from patients with PCD and ovarian or breast cancer reacted with proteins of 62-64 kDa and 34-38 kDa [8]. The 62-64 kDa band was later identified as CDR2 [9], while the 34-38 kDa band was identified as CDR1 [6]. CDR1 is a 262 amino acid long protein with an estimated molecular weight of 34 kDa that was first identified in a patient with PCD and adenocarcinoma of the breast [6, 7]. The *CDR1* gene has a single exon and is mapped to the X chromosome [10, 11]. A mouse orthologue has also been described [11]. The protein consists of 34 inexact repetitive hexapeptides with a core of the acidic amino acids glutamate (E) and aspartate (D) flanked by other amino acids [6]. This repetitive pattern accounts for more than 50% of the human CDR1 sequence (www.uniprot.org, #P51861). The amino acid distribution gives CDR1 a polarized composition with a negatively charged N-terminus and a positively charged C-terminus.

*CDR1* mRNA is highly expressed in human, rabbit, and mouse cerebellum and cerebral hemisphere cortex [6]. Low *CDR1* mRNA levels are found in human lung, kidney, and heart muscle [6]. *CDR1* mRNA is also highly expressed in neuroblastoma and renal cell carcinoma, but has not been found in breast and ovarian cancer cell lines [6]. Furneux et al. found that CDR1 protein was specifically expressed in Purkinje cells in the cerebellum and that CDR1 antibodies recognized a band of approximately 34 kDa in a patient with PCD and breast tumor, but not in a patient with breast tumor without PCD [7]. The association between CDR1 and ovarian and breast cancer and PCD is not well established, but dysregulation of CDR1 has been associated with other neurological diseases like Huntington’s and Alzheimer’s disease [12, 13], and various cancers like prostate cancer and glioblastoma [14, 15]. Recently, it was reported that the antisense strand of *CDR1* (*CDR1-AS*, also known as *ciRS-7*) is transcribed into a circular exonic RNA with a *miR-7* sponge function that stabilizes *CDR1* mRNA [16-18].

So far very little is known about the distribution and function of CDR1, as well as the prevalence of these antibodies in patient sera. In this study, we have therefore characterized CDR1 by examining the expression of CDR1 protein in cerebellum, ovarian cancer and cancer cell lines. We have also studied the prevalence of CDR1 antibodies in sera from patients with Yo antibodies.

## RESULTS

### CDR1 antibody specificity and CDR1 expression in various tissues

**Figure 1:**
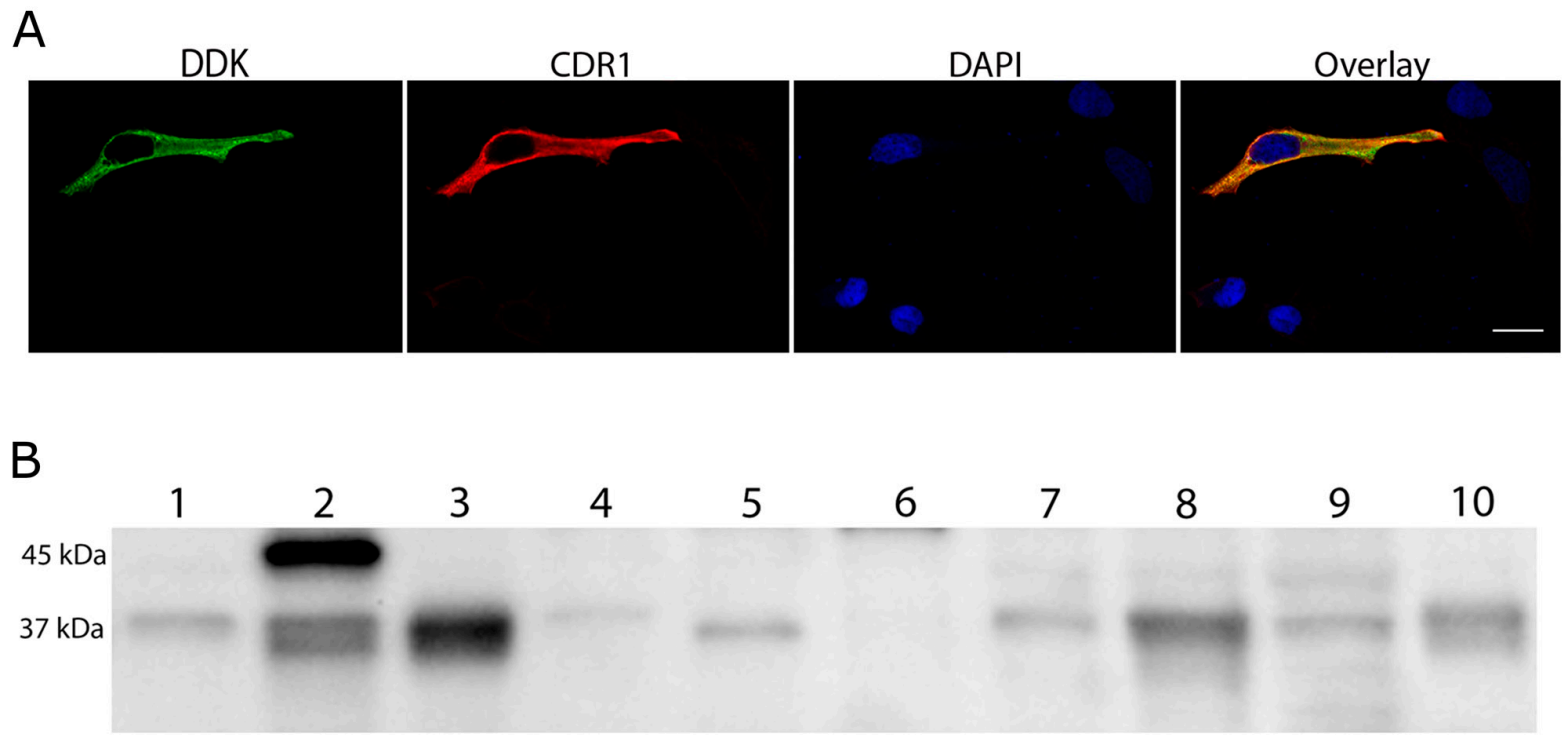
CDR1 antibodies recognize cells overexpressing myc/DDK-CDR1 **(A)** Chicken CDR1 antibody (red) binds specifically to cells overexpressing myc-/DDK-CDR1. Anti-DDK (green) was used to identify transfected cells. The nucleus was visualized with DAPI (blue). Scale bar 20 μm. **(B)** Western blot with CDR1 rabbit antibody. Lane 1: Untransfected HeLa cell lysate, 4 μg; Lane 2: Lysate of HeLa cells overexpressing CDR1, 4 μg; Lane 3: Human cerebellum, 15 μg; Lane 4: Normal ovary, 20 μg; Lane 5: Ovarian tumor, 20 μg; Lane 6: Normal breast, 20 μg; Lane 7: Breast tumor, 20 μg; Lane 8: BT474 lysate, 20 μg; Lane 9: OVCAR 3 lysate, 15 μg; Lane 10: Human Purkinje cell lysate, 5 μg.

The CDR1 antibodies were co-localized with overexpressed myc/DDK tagged CDR1 in HeLa cells (Figure 1A), and specifically recognized myc/DDK-CDR1 in HeLa lysate from cells overexpressing myc/DDK-CDR1, as shown by western blot (Figure 1B). Overexpression of CDR1 in HeLa cells gave a strong band of approximately 45 kDa and a weaker double-band of approximately 37 kDa. The 37-kDa isoform was also present in untransfected HeLa lysates. Incubation with DDK antibody, which only detects the DDK tag of the protein, confirmed that both bands were myc/DDK-CDR1 (Supplementary Figure 1A). The CDR1 antibodies did not recognize recombinant CDR2 and CDR2L proteins in western blot (Supplementary Figure 1B).

The 37-kDa isoform of CDR1 was abundant in the cerebellum and Purkinje cell lysates, as well as in breast (BT474) and ovarian (OVCAR3) cancer cell lysates. This CDR1 isoform was also observed in ovarian and breast tumor lysates, but not in lysates from normal ovaries or normal breast.

### CDR1 expression in cerebellum

**Figure 2:**
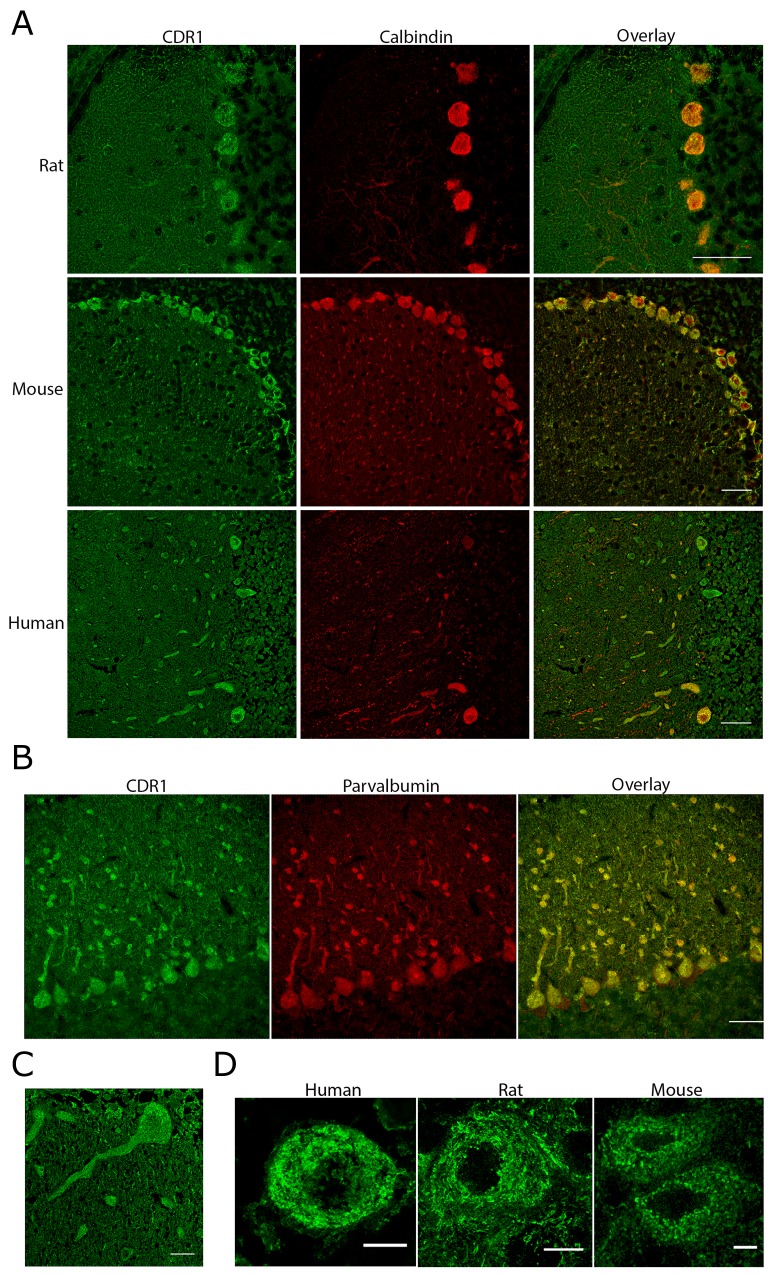
CDR1 is expressed in soma and dendrites of Purkinje cells in rat, mouse, and human **(A)** Chicken CDR1 antibody binds strongly to the Purkinje cells in rat, mouse, and human cerebellum. Staining of interneurons in the molecular layer is also observed. Scale bar 50 μm. **(B)** Co-staining of rat cerebellum with parvalbumin and CDR1 shows that CDR1 is expressed in interneurons of the molecular layer. Scale bar 50 μm. **(C)** CDR1 expression in human Purkinje cell soma and dendrites. Scale bar 20 μm. **(D)** CDR1 is expressed in distinct ring-like structures in the soma of Purkinje cells in human, rat, and mouse. An inner ring is localized just outside the nucleus and a second ring is localized close to the plasma membrane. Scale bar 10 μm. All images are taken using a Leica SP2.

The CDR1 antibodies stained Purkinje cells in human, rat, and mouse cerebellum (Figure 2A). Co-staining with calbindin, a Purkinje cell marker [19], and CDR1 antibody confirmed that CDR1 was expressed in the cytosol and dendrites of Purkinje cells in human, rat, and mouse. Interneurons in the molecular layer of human and rat cerebellum were also stained for CDR1. Co-staining with anti-parvalbumin, a marker for stellate and basket cells [19], showed that CDR1 was also present in these cells (Figure 2B). In addition, CDR1 was found in large neurons in the dentate nucleus (data not shown). CDR1 was expressed in Purkinje cell cytoplasm and dendrites (Figure 2C). A similar staining pattern of Purkinje cell soma and dendrites was seen in human, rat, and mouse. In the Purkinje cell soma, CDR1 was localized as ring-like structures in close proximity to the nucleus and the cell membrane (Figure 2D).

### CDR1 expression in ovarian tumors

**Figure 3:**
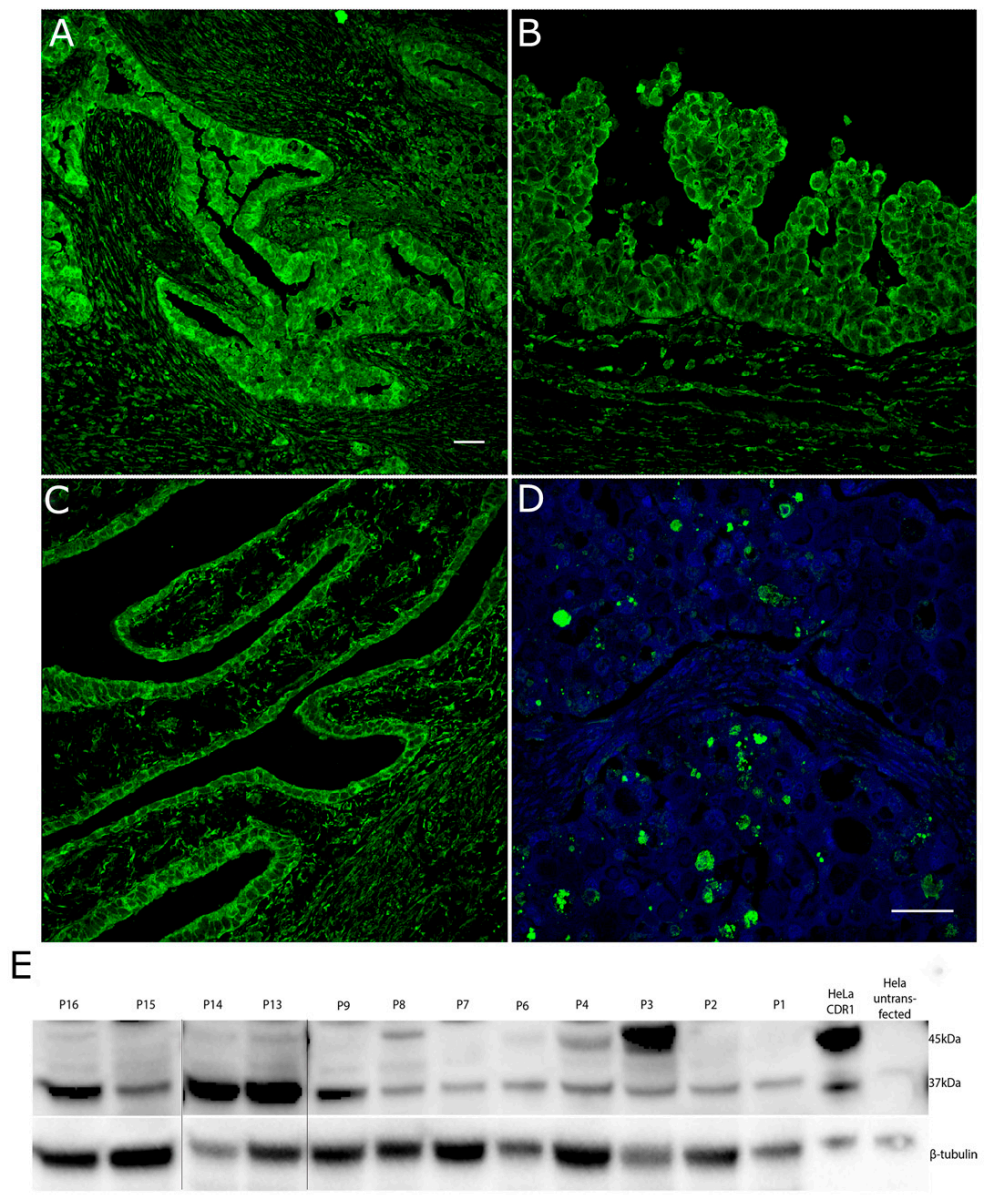
CDR1 is strongly expressed in solid glands in ovarian tumor tissue **(A)** An ovarian tumor section from a patient with high grade serous carcinoma (HGSC), FIGO stage IIIc, PCD and Yo antibodies showed strong CDR1 staining in tumor glands. Surrounding stromal cells showed weaker staining. **(B)** In a section from a patient with HGSC, FIGO IIIc, no PCD, and no Yo antibodies, cancer cells in the epithelial lining stained strongly for CDR1. **(C)** CDR1 staining of the epithelial lining was also observed in a section from a patient with HGSC of unknown differentiation that had no Yo antibodies and no PCD. **(D)** Strong staining for CDR1 was also observed in the tumor from a patient with clear cell ovarian carcinoma, FIGO stage 1A. Nuclei were stained with Dapi. **(E)** Western blot of human ovarian lysates. All lysates expressed the 37-kDa version of CDR1, while a 45-kDa isoform was present in three lysates (P3, P4 and P8). β-tubulin was used as loading control. The image is merged from two gels run in the same setup. Merged sites are shown as black lines. Image a) was taken with 20x objective and images b), c), and d) were taken with a 40x objective on Leica SP2. Scale bar 50 μm.

CDR1 staining was observed by immunofluorescence in all 16 patients with ovarian cancer (Table 1). The staining was specifically strong in the tumor glands (Figure 3A) and in cancer cells along the epithelial lining (Figure 3B and 3C). Strong CDR1 staining was also seen in the clear cell carcinoma (Figure 3D). Normal rat ovaries showed no CDR1 expression (data not shown).

**Table 1:** Clinical characteristics of 16 patients with ovarian cancer

Patient/Age	PNS	Anti-body	Histology	Figo Stage	Differentiation	IF	WB
P1 / 60	PCD	Yo	HSGC	3A	N.D.	+	37 kDa
P2 / 78	-	Yo	HSGC	3B	N.D.	+	37 kDa
P3 / 58	-	Yo	HSGC	4	Well	+	37 kDa, 45 kDa
P4 / 76	PCD	Yo	HSGC	3C	Moderately	+	37 kDa, 45 kDa
P5 / 78	PCD	Yo	HSGC	3C	N.D.	+	Not available
P6 / 65	PCD	Yo	HSGC	3C	Poorly	+	37 kDa
P7 / 74	-	CDR2L	Carsino-sarcoma	3C	N.D.	+	37, kDa
P8 / 72	-	CDR2L	HSGC	3C	Moderately	+	37 kDa, 45 kDa
P9 / 57	-	CDR2L, Zic4	HSGC	4	Poorly	+	37 kDa
P10 / 54	-	CDR2L	Clear cell	1A	N.D.	+	Not available
P11 / 69	-	CDR2L	HSGC	3C	Poorly	+	Not available
P12 / 56	-	-	HSGC	3C	Poorly	+	Not available
P13 / 78	-	-	HSGC	4	N.D.	+	37 kDa
P14 / 69	-	-	HSGC	1A	Poorly	+	37 kDa
P15 / 64	-	-	HSGC	3C	Poorly	+	37 kDa
P16 / 57	-	-	HSGC	3C	Poorly	+	37 kDa

Six patients had Yo antibodies, 4 of these had paraneoplastic cerebellar degeneration (PCD). In 5 patients CDR2L antibodies had been detected previously [4, 33], but these patients had no neurological symptoms. Five patients had no neurological symptoms or known antibodies. IF= immunofluorescence. WB= western blot. (-)= no neurological symptoms / no antibodies. HSGC=High grade serous ovarian cancer. N.D.= not determined.

CDR1 expression was analyzed by western blot in tumor lysates available from 12 of the 16 patients with ovarian cancer (Figure 3E). All patients had a band around 37 kDa, while 3 patients (P3, P4 and P8) also had a band around 45 kDa. The three patients with a 45-kDa band were in FIGO stage 3C or 4 with metastatic tumors.

### Expression of CDR1 in cancer cell lines

**Figure 4:**
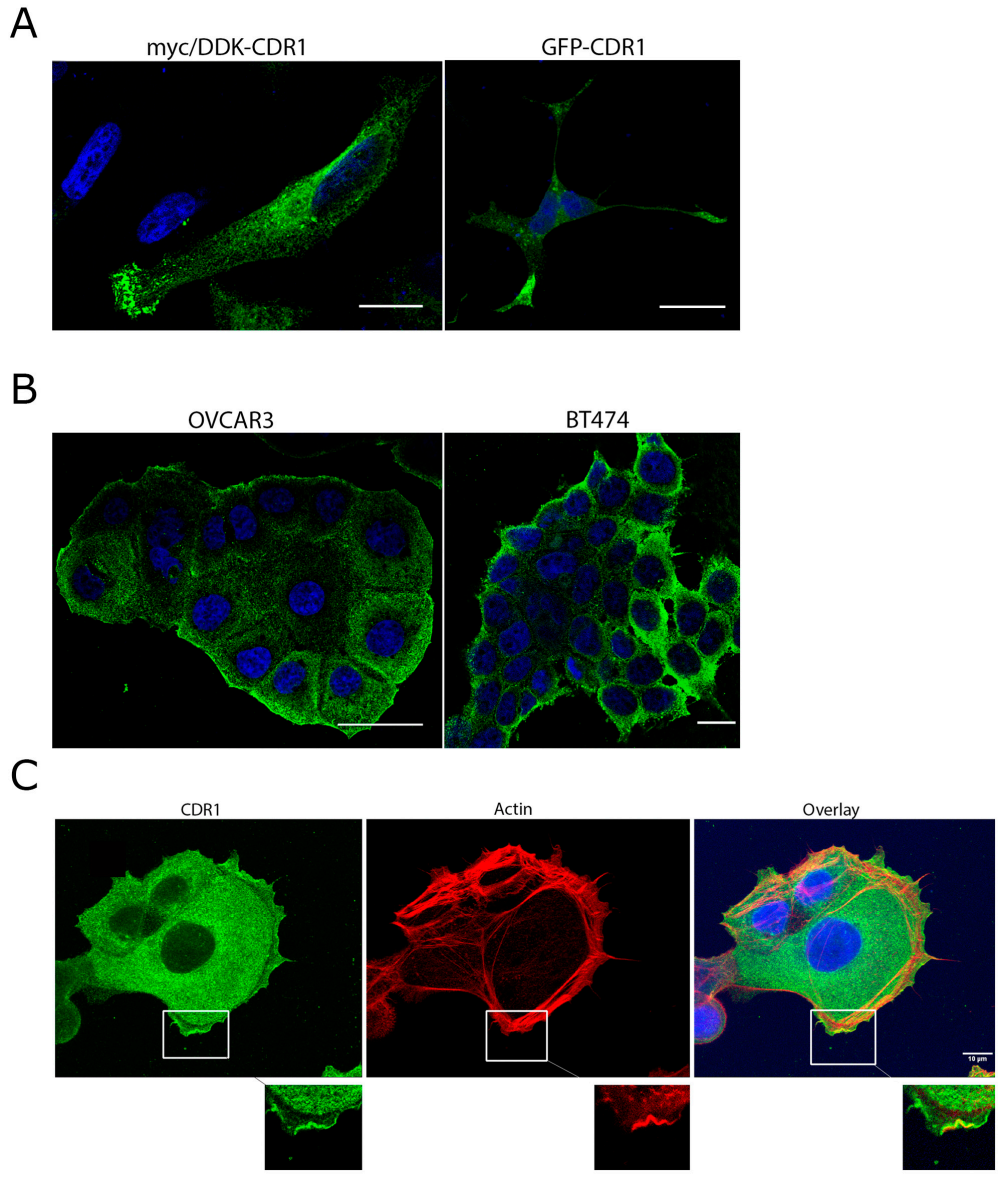
CDR1 expression in cancer cell lines **(A)** Overexpression of myc/DDK-CDR1 in transfected HeLa cells: Left image: Hela cells transfected with myc/DDK-CDR1 (18 h transfection) and stained with anti-myc (green). CDR1 accumulates in the front-line of the cell. Right image: Cells transfected with CDR1-GFP (24 h transfection). CDR1 accumulates in the protrusions of the cell. Scale bar 20 μm. **(B)** Both OVCAR3 and BT474 express CDR1 endogenously. The staining is stronger in the cells making the outer border of the cell colony. **(C)** Endogenous CDR1 expression (green) in OVCAR3 cells co-stained with the actin marker phalloidin (red). CDR1 is strongly expressed close to the plasma membrane and in the cytoplasm. CDR1 is not present in an area between the cortical actin and the outer membrane ruffles. However, CDR1 is expressed in the membrane protrusions where actin is involved in lamellipodia/filopodia formation. Main images are flattened images of the stack. Boxed areas are zoom in on a single section if the confocal plane. CDR1 co-localizes with actin in the membrane ruffles. Scale bar 10 μm. Nuclei are stained with DAPI (blue).

Intracellular distribution of CDR1, examined by overexpressing myc/DDK-CDR1 or GFP-CDR1 in HeLa cells, showed cytoplasmic localization with some staining close to the nucleus. However, CDR1 accumulated in a polarized pattern at one of the cell edges or in outgrowths of the cells (Figure 4A). Endogenous expression of CDR1 in the ovarian cancer cell line OVCAR-3 and the breast cancer cell line BT474 showed that CDR1 was localized to the cytoplasm and plasma membrane. No staining was observed in the nucleus. The staining was specifically strong in the plasma membrane in the cells along the outer surface of the cell colony, whereas the cells in the middle with less room for expansion showed little CDR1 expression (Figure 4B). Co-staining of OVCAR-3 cells with CDR1 and the actin marker phalloidin showed strong CDR1 staining in membrane ruffles in areas where actin protrusions was observed. CDR1 staining was also observed in the cytoplasm close to the cortical actin filaments, but was absent in the area between the cortical actin and the membrane ruffle. Dotted staining close to the nucleus was also observed (Figure 4C).

### Detection of CDR1 antibodies in sera

**Figure 5:**
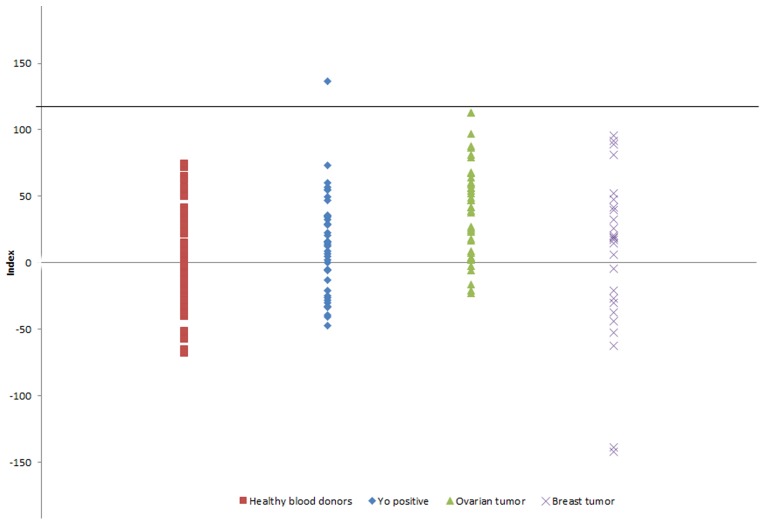
CDR1 indexes in patient sera from patients with PCD and Yo antibodies, patients with ovarian cancer without neurological symptoms, and patients with breast tumor without neurological symptoms Only one patient with PCD and Yo antibodies was positive for CDR1 antibodies. Black line indicates cut-off.

The prevalence of CDR1 antibodies were tested by an *in vitro* transcription-translation and immunoprecipitation assay in sera from 40 patients with Yo antibodies (Supplementary Table 1), and in sera from 40 patients with ovarian cancer and 25 patients with breast cancer without onconeural antibodies. The index distribution of the sera from the 50 healthy blood donors used as controls was used to determine background levels and cut-off. The cut-off of detection determined from the average index of the 50 blood donors (+ 3 standard deviations) was set to 114. CDR1 antibodies were detected in only one serum with an index of 136 (Figure 5). This patient was Yo positive and had PCD and ovarian cancer 2 (patient 2 in Supplementary Table 1). The patient was a 55-year-old woman who developed PCD 20 months before a pelvic tumor was discovered. Autopsy revealed ovarian adenocarcinoma with disseminated peritoneal tumor growth, and metastasis to para-aortic lymph nodes and skeletal bones [21]. The serum sample that was analyzed was taken at the time of cancer diagnosis. Tumor tissue from this patient was not available for our study.

## DISCUSSION

We found that CDR1 is expressed in human, mouse and rat Purkinje cells, as well as in interneurons in the molecular layer. This is in line with previous reports where CDR1 was detected in Purkinje cells in human cerebellum [7]. This is supported by BioGPS, which shows that *CDR1* mRNA is highly expressed in cerebellum, but not in other normal tissue (biogps.org). We found that CDR1 was expressed as ring-like structures throughout the cytoplasm of the Purkinje cells, especially around the nucleus and close to the plasma membrane. Whether CDR1 is a membrane protein or a cytoplasmic protein that resides in or close to organelle membranes like the endoplasmic reticulum remains to be elucidated.

We also found CDR1 expression in the Purkinje cell dendrites. Interestingly, the circular RNA *CDR1-AS* localizes to the dendrites of neurons in the somatosensory cortex in mice [17]. As *CDR1-AS* is important for stabilizing *CDR1* mRNA [18], this implies that *CDR1* mRNA is translated in the dendrites. mRNAs locally translated in dendrites code for proteins involved in dendritic spine morphology, dendritic branching, and neuronal excitability [20]. Therefore, it is likely that the localization of CDR1 in the dendrites is important for dendritic morphology and branching. CDR1 has an estimated molecular weight (MW) of 31.3 kDa based on amino acid sequence. Previous reports have suggested a MW of 34 kDa based on western blot [7]. Our results showed that cells overexpressing CDR1 produce a strong band of approximately 45 kDa and a weaker double-band of approximately 37 kDa. These western blot findings can partly be explained by amino acid composition and cellular localization. Acidic proteins that contain a high percentage of glutamate and aspartate, which is the case for CDR1, migrate more slowly on SDS-PAGE than their predicted MW [21]. CDR1 consists of 34 inexact repetitive hexamer amino acid sequences with a core of glutamate and aspartate. Glutamate and aspartate constitutes 22.5% of the total amino acid composition of human CDR1. Further, our findings suggest that CDR1 is associated with cell membranes, and it has been shown that detergent binding results in anomalous migration of membrane proteins on SDS-PAGE [22].

*CDR1* mRNA is highly expressed in the cerebellum and has also been identified in neuroblastoma, renal cancer and prostate cancer cell lines, but was not found in breast and ovarian cancer cell lines [6]. CDR1 protein has previously been found in Purkinje cell extract and in breast tumor lysate from a patient with PCD, but not in breast tumor lysate from a patient without PCD [7]. In this study, CDR1 was not observed in cortical neurons, normal liver, small cell lung carcinoma or normal ovary [7]. We found, however, that CDR1 was expressed in both ovarian and breast cancer lysates, regardless of PCD status, but not in normal ovarian or breast tissue. Furneaux et al [7] used a CDR1 antibody against amino acid 149-165, while our CDR1 antibodies were directed against amino acid 99-129. This increased length of the CDR1 peptide could improve antibody sensitivity, and could explain why we found CDR1 also in tumors from patients without PCD.

We found a 37 kDa and a 45 kDa band in HeLa cells overexpressing CDR1. The 37-kDa CDR1 isoform was strongly expressed in cerebellum and Purkinje cell lysates, as well as in the breast cancer cell line BT474 and the ovarian cancer cell line OVCAR3. Dropcho et al. did not find *CDR1* mRNA expression in breast or ovarian cancer cell lines [6], but it is possible that they used other cell lines with lower *CDR1* expression. This is in accordance with the BioGPS (www.biogps.org) which shows that *CDR1* is expressed at various levels in various cell lines; for example OVCAR3 express above average levels of *CDR1*, while other ovarian cancer cell lines express less. Since we found the 37-kDa band in untransfected HeLa lysates, in ovarian and breast tumor lysates and in lysates from all ovarian cancer patients examined, it is likely that this is the CDR1 isoform that is naturally expressed in the body. The 45-kDa band was not present in any of the neuronal lysates tested, but we found this band in 3 ovarian cancer patients. The 45-kDa isoform was the dominant form of CDR1 in the cells overexpressing CDR1, which could be due to post-transcriptional modifications like ubiquitination or glycosylation. All tumors were CDR1 positive by immunofluorescence, and the staining was similar in the ones that only expressed the 37-kDa isoform and those that expressed both isoforms.

We found that CDR1 was expressed in a polarized pattern towards one of the edges in the cancer cells in culture. This polarized expression could be seen in single HeLa cells overexpressing CDR1, and in colonies of cancer cells that express CDR1 endogenously. Such polarization of proteins have been associated with cell migration [23], which involves actin and microtubules [24]. We found that CDR1 was expressed in areas of actin protrusions in the plasma membrane, indicating a role for CDR1 in the generation of filopodias or lamellipodias. That CDR1 may have a role in cell migration is also supported by knock-out experiments of *CDR1-AS* in HEK293 cells which resulted in reduced migration rate [16], possibly due to destabilization of *CDR1* [18]. Furthermore, upregulation of *CDR1-AS* is associated with increased risk of hepatic microvascular invasion in hepatocellular carcinoma [25]. Also, the dynein associated protein ZMYND10 is a potential interaction partner for CDR1 based on yeast two-hybrid assays [26]. Since mutations in ZMYND10 are associated with decreased ciliary function [27], this also supports the hypothesis that CDR1 may have a role in cell migration.

We detected serum CDR1 antibodies in only one of 40 Yo-positive sera. The patient had PCD and ovarian cancer, while a previous report found CDR1 antibodies in a patient with PCD and breast cancer [6]. This indicates that CDR1 antibodies can be associated with PCD in both ovarian and breast cancer. However, why CDR1 antibodies are so rarely found in cancer patients is not known. Our patient had an advanced ovarian cancer with metastases and whether the presence of serum antibodies reflects upregulation of CDR1 in advanced tumors remains to be shown. Such an association could be supported by our findings in cell culture which suggest that CDR1 may have a function in cell migration,

In summary, we found that CDR1 is expressed in cerebellar tissue, as well as in breast and ovarian tumors and cancer cell lines, but not in normal ovarian or breast tissue. The presence of CDR1 in ovarian cancer was not associated with PCD and CDR1 antibodies were only found in serum from one patient with PCD and ovarian tumor with metastases. Therefore, CDR1 is probably not a marker for PCD. We found that CDR1 was localized to soma and dendrites of Purkinje cells, whereas in cancer cells CDR1 was localized to protrusions of the plasma membrane. This indicates that CDR1 may be associated with cell differentiation and migration.

## MATERIALS AND METHODS

### Antibodies

Chicken and rabbit CDR1 antibodies were made by immunizing the animals with a CDR1 peptide sequence (amino acids 99-129 (Eurogentec). Chicken anti-CDR1 IgY was affinity purified by Eurogentec; the stock solution was at a concentration of 1 μg/μl. Pre-immune chicken IgY was received as egg yolks collected from the hens before immunization with the CDR1 peptide. IgY was purified from the egg yolks with Pierce Chicken IgY Purification kit (Thermo Fisher) according to the manufacturer’s instructions. The concentration was adjusted to 1 μg/μl and used in similar dilutions as the chicken CDR1 antibody. The chicken CDR1 antibody was used for immunostaining, and the rabbit CDR1 was used for western blot and in the *in vitro* transcription-translation assay. We used chicken antibodies as these could be used in double staining with other rabbit antibodies. Furthermore, chicken antibodies elicit a strong immune response against mammalian proteins. The chicken and rabbit antibodies gave a similar staining pattern in immunofluorescence and western blot, but less background was observed for the chicken antibody than for the rabbit antibody in immunofluorescence studies.

Mouse anti-DDK (#TA50011) and anti-myc (#TA15021) antibodies were purchased from OriGene, mouse anti-Calbindin D28K (#C9848) from Sigma-Aldrich, sheep anti-parvalbumin (#AF5058) from R&D Systems, Alexa Fluor 594 phalloidin and Alexa fluor 488 or 594 conjugated goat secondary antibodies were purchased from Thermo Fischer. All antibodies were used in accordance with manufacturer’s instructions.

### Cell lines

All cell lines were purchased from and authenticated at American Type Culture Collection (ATCC). HeLa cells (#ATCC-CCL-2) were grown in DMEM supplemented with 10% FCS (Sigma), 1% L-glutamine (Life Technologies, #25030081), and 1% penicillin/streptomycin (Sigma, P4333). Cells were grown in 75-cm^2^ tissue culture flasks (VWR, #734-2314).

OVCAR-3 cells (ATCC, #HTB-161) were grown in RPMI-1640 (Life Technologies, #61870044) containing 0.01 mg/ml insulin (Life Technologies, #61870044), 20% hi-FBS (Life Technologies, #10500064), 0.45% D-Glucose (Sigma, #G8769), 1% sodium pyruvate (Life Technologies, #1130070), and 1% penicillin/streptomycin.

BT474 cells (ATCC, #HTB-20) were grown in RPMI-1640 supplemented with 0.01 mg/ml insulin, 10% hi FBS, and 1% penicillin/streptomycin. OVCAR-3 and BT474 cells were grown on 22-mm^2^ glass coverslips coated with poly-D lysine (Neuvitro, #GG-22-PDL).

### Overexpression of CDR1

Plasmids encoding full-length myc-DDK tagged CDR1 (#RC217579) or CDR1 with a C-terminal GFP tag (#RG217579) were purchased from OriGene, amplified in *E. coli* and purified with QIAGEN Plasmid Midi Kit. The purified plasmid was used for overexpression of CDR1 in HeLa cells.

For transfection, cells were seeded on poly-L-lysine coated cover glasses and grown in 24-well Nunclon delta multidishes. Cells were transfected at approximately 90% confluency using Lipofectamine 3000 Reagent (Thermo Fisher) according to the manufacturer’s instructions. The media of the cells was changed to a media without antibiotics before transfection. For each well to be transfected, 0.5 μg DNA was mixed with 25 μl OptiMEM (Life Technologies, #31985,) and 1 μl P3000 reagent in a tube. In another tube 1 μl Lipofectamine 3000 was added to 25 μl OptiMEM. The two tubes were mixed and incubated in room temperature for 5 minutes before the transfection mix was added to the cells. For cell imaging, the cells were fixed in a PBS solution containing 4% paraformaldehyde (PFA) and 4% sucrose for 20 min.

For protein purification, HeLa cells were grown on 10-cm^2^Nunclon delta surface dishes for 2 days before transfection. The transfection protocol was up-scaled to suit the size of the growth plate. After transfection cells were incubated for 18 h. Before protein purification the cells were washed twice in cold PBS and spun down. A Millipore total protein extraction kit (#2140) was used according to the manufacturer’s instructions for protein purification. Protein concentration was determined using a Pierce BCA protein assay (Thermo Fisher, #23225).

### Ovarian cancers

We investigated CDR1 expression in tumors from 16 patients with ovarian cancer by immunofluorescence. Fourteen of the 16 patients had high grade serous carcinoma (HGSC), FIGO stage 1A – 4, one patient had clear cell carcinoma (FIGO stage 1A), and one patient had carcinosarcoma (FIGO stage 3C). Six of the patients had Yo antibodies, and four of these had been diagnosed with PCD. Since we did not have normal human ovaries, normal ovaries from perfused rats served as a negative control.

### Immunofluorescence

Rat and mouse cerebellums from PFA-perfused animals were post-fixed in 4% PFA for 2 days, and incubated in an 18 % sucrose/PBS solution for 1 day before the cerebellums were snap-frozen into bolts in ice-cold isopentane. The bolts were frozen at -80°C, and 10-μm sections were cut on a Leica CM1960 cryostat for immunofluorescence studies.

Human paraffin-embedded tissues were cut into 5-μm sections. To prevent tissue detachment, slides were incubated at 60°C for 30 minutes. Sections were deparaffinized in Histolab Clear (#14250) before hydration in decreasing grades of alcohol.

For epitope retrieval all sections were boiled in a 2100 Retriever with Diva decloaker buffer (Histolab, #BC-DV2004) as described by the manufacturer. Slides were rinsed in distilled water. For both sections and cells, autofluorescence was quenched by a 5-minute incubation in 50 mM NH_4_Cl. Cells were permeabilized in 0.1% Triton X-100/PBS for 5 minutes, before blocking by a 1-hour incubation in a 1/3 Sea Block/PBS solution. Sections were blocked and permeabilized in a 1:3 SEA Block:PBS solution containing 1% Triton X-100. Sea Block (Thermo Fisher, #37527) was used as it is a very efficient blocking reagent for chicken antibody. The sections or cells were then incubated overnight with primary antibodies. Chicken antibodies were diluted 1:1000 or 1:2000 in 1:10 Sea Block:PBS containing 0,3% Triton X-100. In some instances a dual staining with additional antibodies was performed. In these cases, on the next day the slides were washed three times in PBS, before 2-hour incubation with fluorescently labelled secondary antibodies. Slides were mounted in Prolong Gold with Dapi (Life Technologies, #P36941) and examined by confocal imaging. Images were taken with Leica Confocal SP2 or SP5 microscopes with 40×, 63×, or 100× objectives.

### Western blot

Human lysates were purchased from ProSci. Total protein extraction kit (Millipore) was used according to the manufacturer’s instructions to make lysates from HeLa, OVCAR-3 and BT474 cells. Protein concentrations were determined using a Pierce BCA protein assay kit. Lysates of fresh frozen ovarian cancer tissue were prepared at the Proteomics Unit at the University of Bergen, (www.biochem.mpg.de/226356/FASP). Recombinant CDR2 and CDR2L proteins were produced as described elsewhere [4], and 1 μl protein was added to each well.

For gel electrophoresis, 20 μg protein from Prosci lysates or approximately 50 μg protein from the ovarian cancer lysates were loaded into each well with 3 μg untransfected HeLa lysate and 3 μg myc/DDK-CDR1-overexpressing HeLa lysate, used as negative and positive controls respectively, to determine the size of CDR1. The proteins were separated on precast 12% Mini-Protean TGX gels. Proteins were blotted onto a PVDF membrane using a Trans Blot Turbo Transfer Pack on the Trans-blot Turbo system, all from Biorad. Nonspecific binding was blocked by 1-hour incubation in PBS containing 5% non-fat dry milk, before overnight incubation at 4°C with a rabbit CDR1 antibody diluted 1:1000 in PBS-Tween (0.05%) with 0.5% dry milk, followed by 1-hour incubation with secondary antibody swine anti-rabbit HRP antibody (DAKO, #P0217) diluted 1:2000 in PBS-Tween with dry milk. β-tubulin (Sigma-Aldrich, #T4026) was used as loading control. The blots were developed using the chemiluminescence, Clarity western ECL substrates, and imaged on Chemidoc XRS+ (BioRad).

### *In vitro* transcription-translation (ITT) and immunoprecipitation (IP)

CDR1 cDNA was PCR amplified from the vector pCR-4 TOPO (ATCC) into a pET-100/D-TOPO vector (Thermo Fisher). ITT and IP were performed as previously described [28-32]. In brief ITT was performed using the TNT coupled reticulocyte lysate system (L4610, Promega) to produce [^35^S]-methionine-labeled CDR1 protein by adding [^35^S]-methionine (# NEG709A, PerkinElmer) to the reaction. MultiScreen 96-well plates with filter bottom (#MABV N12; Millipore) were used for IP. Each well was preincubated with 200 μl of buffer A (150 mmol/l NaCl, 20 mmol/l Tris-HCl and 0.01 % azide, pH 8.0) for 1 hour at room temperature. Buffer A was discarded and the wells were then blocked with 1% BSA (Sigma) in buffer A for 2 hours at room temperature and washed twice with 0.05% Tween-20 in buffer A. Finally, the wells were washed once with buffer B (0.1 % BSA and 0.05% Tween-20 in buffer A).

[^35^S]- labelled CDR1 protein (30 000 cpm/well) and sera diluted 1:10 in incubation buffer (20 mmol/l Tris–HCl, 150 mmol/l NaCl, 0.1% BSA 0.15% Tween-20 and 0.001% azide, pH 8.0) were incubated at 4°C overnight. The next day, 50 μl of a 50% slurry of resuspended Protein-A Sepharose (GE Healthcare) in incubation buffer was added to each well of the MultiScreen plates followed by the addition of the immune complexes. The plates were then incubated on a shaking platform for 45 min at 6°C, washed, and left to dry overnight. Finally, 20 μl of scintillation fluid (Microscint 20; Perkin Elmer) was added to each well, and the amount of radiolabeled immunoprecipitate was measured in a betacounter (Topcount NXT microplate scintillation and luminescence counter; Perkin Elmer). Each patient serum sample was run in triplicate, and the mean value of these was used. The results were expressed as an index: (cpm sample – cpm negative control) / (cpm positive control – cpm negative control) × 1000. Pooled sera from 100 blood donors were used as negative control, and polyclonal rabbit CDR1 antibody was used as positive control.

CDR1 antibody index was measured in sera from 40 patients with Yo antibodies, of which 30 had PCD, 5 patients had breast cancer and 25 had ovarian cancer. The type of cancer and/or PCD status was unknown for 10 patients. The 40 Yo patients with Yo antibodies were selected from samples sent for routine analysis for onconeural antibodies at the Neurological Research Laboratory, Haukeland University Hospital. Positive samples are stored in a Research Biobank. In addition 40 patients with ovarian and 25 patients with breast cancer without any onconeural antibodies or neurological symptoms were screened. Presence of onconeural antibodies were determined by a commercial immunoblot (PNS 11 Line Assay, Ravo Diagnostika GmbH, #PNS11-003/48) containing recombinant CDR2 protein. Seronegative ovarian cancer patients were randomly selected from ovarian cancer patient samples in the Bergen Gynecologic Cancer Biobank, Haukeland University Hospital. Seronegative breast cancer samples were included from control material from the Research Biobank at the Neurological Research Laboratory. Sera from 50 healthy blood donors at the Blood bank, Haukeland University Hospital were tested to determine the natural variation in serum samples and to calculate cut-off. All sera had been tested negative for onconeural antibodies. Cut-off was calculated as the mean index of the 50 blood donors (+ 3 standard deviations). Patient samples with an index greater than the cut-off value were considered positive. Positive results were rerun three times, and each repetition had to yield an index above cut-off to be considered positive.

## ETHICAL ISSUES

All procedures performed involving human participants were approved by The Regional Committee for Health and Medical Research Ethics (2014/1066) and were in accordance with the Declaration of Helsinki (1964) and its later amendments or comparable ethical standards. Informed consent was obtained from living patients included in the study. Patient sera used were from the Paraneoplastic neurological disease biobank (#484) and approved by the Regional Committee for Medical and Health Research Ethics in Western-Norway, Diagnostic markers of cancer (188.05). Procedures involving animals were performed according to the National Institutes of Health Guidelines for the Care and Use of Laboratory Animals Norway (FOTS 20135149/20113133).

## SUPPLEMENTARY MATERIALS FIGURE AND TABLE


