# Supplementary material for: Expression of the onconeural protein CDR1 in cerebellum and ovarian cancer
Source: Oncotarget. 2018 May 8;9(35):23975–86. doi: 10.18632/oncotarget.25252 (PMC5963614; doi:10.18632/oncotarget.25252)
Supplement: Supplementary file 1 [file oncotarget-09-23975-s001.pdf]

Expression of the onconeural protein CDR1 in cerebellum and ovarian cancer

SUPPLEMENTARY MATERIALS

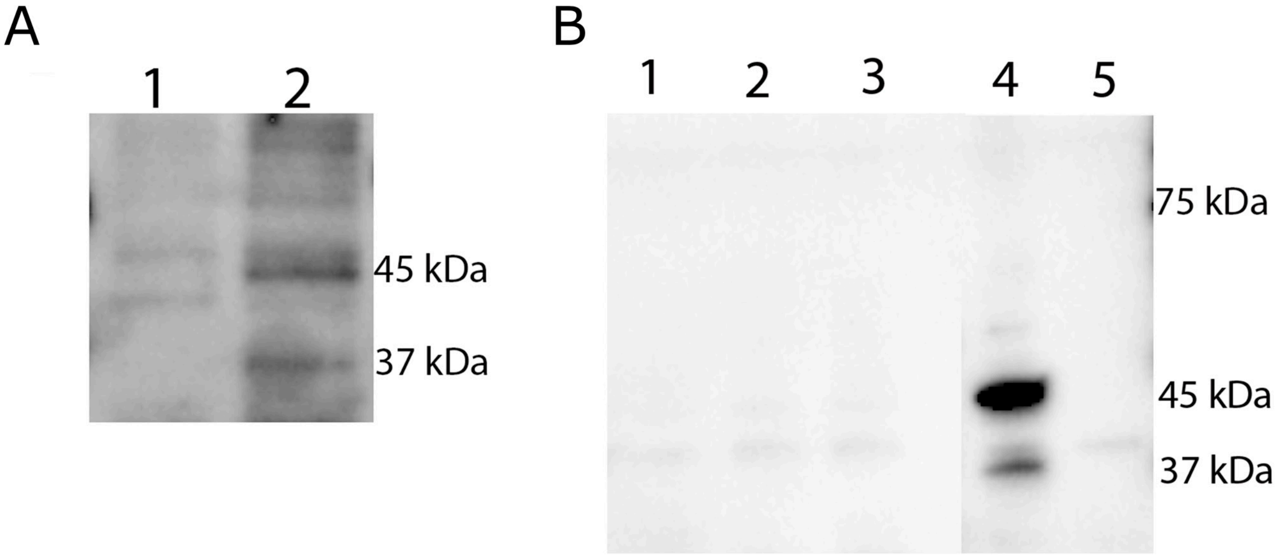

Supplementary Figure 1: (A) Lane 1: Untransfected HeLa Lysate, 10 µg. Lane 2: HeLa lysate overexpressing myc/DDK-CDR1, 10 µg. DDK antibodies recognize two bands of approximately 45 kDa and 37 kDa in HeLa cells overexpressing myc/DDK-CDR1. These bands are not present in untransfected cells. **(B)** Lane 1: Untreated Rabbit reticulocyte lysate 1 µl. Lane 2: Recombinant CDR2L, 1 µl. Lane 3: Recombinant CDR2, 1 µl. Lane 4: HeLa lysate overexpressing myc/DDK-CDR1, 3 µg. Lane 5: Untransfected HeLa lysate, 3 µg. CDR1 Antibodies do not recognize recombinant CDR2 or CDR2L proteins.

Supplementary Table 1: Overview of type of tumor and paraneoplastic syndromes for the 40 patients with Yo antibodies

Patient	Age, sex	Neurological syndrome	Type of cancer
1	81, F	PCD	Ovarian cancer
2	55, F	PCD	Ovarian cancer
3	66, F	PCD	Ovarian cancer
4	57, F	Unknown	Breast cancer
5	59, F	PCD	Tube cancer
6	60, F	PCD	Ovarian cancer
7	78, F	Unknown	Ovarian cancer
8	57, F	PEM	Unknown
9	F, 64	PCD	Breast cancer
10	F, 39	PCD	Ovarian cancer
11	44, F	PCD	Ovarian cancer
12	55, F	PCD	Unknown
13	66, F	PCD	Tube cancer
14	66, F	PCD	Breast cancer
15	52, F	PCD	Ovarian cancer
16	46, F	Unknown	Unknown
17	78, F	PCD	Ovarian cancer
18	70, F	PCD	Tube cancer
19	58, F	PCD	Tube cancer
20	65, F	PCD	Corpus cancer
21	56, F	PCD	Ovarian cancer
22	70, F	PCD	Breast cancer
23	54, F	PCD	Ovarian cancer
24	72, F	PCD	Endometroid cancer
25	58, F	PCD	Ovarian cancer
26	64, F	PCD	Unknown
27	79, F	Unknown	Ovarian cancer
28	63, F	Unknown	Lung metastasis
29	57, F	PCD	Ovarian cancer
30	57, F	PCD	Ovarian cancer
31	70, F	Polyneuropathy	Unknown
32	65, F	PCD	Ovarian
33	54, F	PCD	Ovarian cancer
34	65, F	PCD	Ovarian cancer
35	71, F	PCD	Ovarian cancer
36	68, F	Unknown	Unknown
37	51, M	Unknown	Unknown
38	34, F	Unknown	Unknown
39	81, F	PCD	Ovarian cancer
40	72, F	PCD	Breast cancer

F = female, M = male, PCD = paraneoplastic cerebellar degeneration, PEM = paraneoplastic encephalomyelitis.
